# “Do Good, Have Good”: A Serial Mediation Analysis of CSR with Customers’ Outcomes

**DOI:** 10.3389/fpsyg.2020.00598

**Published:** 2020-04-28

**Authors:** Ishfaq Ahmed, Mian Sajid Nazir, Imran Ali, Arooj Khalid, Muhammad Zeeshan Shaukat, Farooq Anwar

**Affiliations:** ^1^Hailey College of Commerce, University of the Punjab, Lahore, Pakistan; ^2^Department of Management Sciences, COMSATS University Islamabad Lahore Campus, Lahore, Pakistan; ^3^Department of Business Administration, Faculty of Economics and Administration, King Abdulaziz University, Jeddah, Saudi Arabia; ^4^Institute of Quality & Technology Management, University of the Punjab, Lahore, Pakistan; ^5^Faculty of Management Sciences, University of Central Punjab, Lahore, Pakistan; ^6^Lahore Business School, University of Lahore, Lahore, Pakistan

**Keywords:** affective commitment, corporate social responsibility, customer citizenship behavior, developing country, fast food restaurants, service quality

## Abstract

Corporate social responsibility (CSR) is envisaged to offer several outcomes and while customer-specific consequences are unclear or have not obtained due attention, there is a dearth of literature that focuses on perceptual, attitudinal and behavioral outcomes in tandem. Against this backdrop, this study entails the investigation of perceptual (service quality), attitudinal (affective commitment) and behavioral (customer citizenship behavior) outcomes of CSR through a serial mediation mechanism. A total of 994 responses obtained from fast-food restaurants, highlight the fact that CSR influences service quality, affective commitment, and customer citizenship behavior. Moreover, it is witnessed that CSR influences customer citizenship behavior indirectly as well, as the serial mediation mechanism is also proved. The practical and theoretical usefulness of the study is also provided at the end.

## Introduction

Recently a business roundtable covering the CEOs of the top 200 firms concluded that maximization of profits and shareholders’ wealth is no more the basic purpose of a business; rather businesses aim at creating value for suppliers, employees, customers, community and the environment at large (Forbes, 2019; Shah et al., 2019). This paradigm shift pays a lot, since socially responsible businesses have been found outperforming their counterparts (Sarfraz et al., 2018; D’Amato and Falivena, 2019), as CSR investments offer a competitive advantage over rivals (Latif and Sajjad, 2018). The shift is in concordance with the current challenges of global warming and increasing economic disparity (Blowfield and Murray, 2014; Carroll, 2016).

The changing landscape of the business practices and its possible outcomes has beckoned researchers and practitioners to carry out studies focusing on the value of such investments for the business (Sarfraz et al., 2019). Past studies have noticed that CSR can offer various positive outcomes at different levels. For instance, CSR investments pay off in shape of positive attitudinal changes in employees (e.g., job satisfaction, commitment, engagement; Asrar-ul-Haq et al., 2017; Story and Castanheira, 2019), innovativeness and competitiveness (Marin et al., 2017), and better market value (D’Amato and Falivena, 2019). Yet another aspect that has largely gained less attention is customer based outcomes of CSR investments (Marin et al., 2009) and while the empirical literature from developing countries is even scarce (Huang et al., 2019), Pakistan is not an exception to it (Gilal et al., 2019).

Against this backdrop, this study entails the investigation of CSR outcomes in shape of customer citizenship behavior (CCB), which is defined as extra-role (voluntary) customers’ behavior and focuses on helping other customers and providing suggestions to improve products and services (Bartikowski and Walsh, 2011; Tung et al., 2017). When the level of CCB is high in organisations, these organisations are found to be more efficient (Mills et al., 1983) with a better competitive position (Yi et al., 2011). While looking at the link between CSR and CCB, literature shows that there is a dearth of studies focusing on this possible association. Moreover, this study also encompasses the investigation of the mechanism between CSR and CCB through serial mediation of service quality perceptions (perceptual factor) and affective commitment (attitudinal factor) of customers. This mechanism has also not gained much attention in past studies of CSR.

We drew our CSR and CCB mechanism with the help of the attachment theory (AT) (Bowlby, 1969), attribution theory (Heider, 1958), and affect infusion model (Forgas, 1995). For instance, AT highlights that customers, due to some organizational positive factors (here CSR), gets attached with a firm or its products/services and resultantly reciprocates through positive behavior (e.g., CCB). Likewise, attribution theory signifies that individuals develop certain perceptions (and adapt actions accordingly) which are attributed to some internal or external factors. Here CSR being the external factor is believed to influence customers’ perceptions and which in return may make them reciprocate positively (through positive attitudinal and behavioral outcomes). The affect infusion model, on the other hand, proposes that effects (emotions and moods) may influence one’s judgments. As CSR investments by the firm may influence customers at an emotional level, it is perceived that customers’ will think about the brand/firm (judgments) positively (Xie et al., 2017; van Tonder et al., 2018).

Though the proposed mechanism (CSR – SQ – AC – CCB) have not been investigated in the past, the need for such a study has been highlighted in literature (Ahmed et al., 2020). For instance, Engizek and Yasin (2017) highlighted that CSR should be investigated for its attitudinal and behavioral customer outcomes. If we propose the use of both commitment (attitude) and CCB (behavior) outcomes, we may fill this gap. Additionally, past studies have also valued the role of various contextual factors (Anaza, 2014; Choi and Lotz, 2018) and mechanism between those factors (Xie et al., 2017) also, while highlighting the antecedents of CCB, there may exist some explanatory mechanisms that could bring true picture and clarity. The aforementioned calls are answered through considering CSR as a contextual factor and the service quality and affective commitment as an explanatory mechanism. Moreover, Islam et al. (2019) commented that service quality should also be investigated for possible attitudinal and behavioral outcomes, which is also one of the major contributions of the current study.

The following sections of the article cover the theoretical stance and hypotheses development, followed by the methodology adopted to test those hypotheses. Findings and conclusions are later on drawn from the results. In the end, implications are discussed and the conclusion is drawn.

## Theorization and Hypotheses Development

### Theoretical Underpinnings

We drew a CSR and CCB relation based on the affect infusion model (Forgas, 1995), attachment (Bowlby, 1969) and attribution theories (Heider, 1958). These theories help in linking CSR with CCB through service quality and affective commitment. The affect infusion model (AIM; Forgas, 1995) proposes that one’s emotions influence cognitive judgments; and ultimately one’s responses. CSR being the organizational investment aimed at society and its members may influence customers’ emotions positively (Pérez and Del Bosque, 2015; Abbas et al., 2018; Xie et al., 2019), which may foster CCB.

The AT of Bowlby (1969) also explains the association, which assumes that one develops a bond with the firm or its product/service and expresses her roles in response (here CCB as a behavioral response). It is evident in literature that organizational consideration toward society and its other member positively influence the bond between the firm and the customers as they react positively (Zhu et al., 2016; Rodrigues and Costa, 2017). While assuming CSR as the care for society and its members, this study entails that such investment will foster a firm-customer bond and will make customers respond positively with high CCB (e.g., Servera-Francés and Piqueras-Tomás, 2019; Xie et al., 2019). Yet another theoretical premise is based on the Attribution theory (Heider, 1958), which proposes that humans attribute their attitude and behavior to internal or external factors. We propose that CSR as an external cause that may influence customers’ attitudes and behavior toward firms investing in CSR. Past studies also highlight that CSR is perceived to be a favorable external attribute that influences customers positively (Marin and Ruiz, 2007; Lii and Lee, 2012; Plewa et al., 2015).

### Hypotheses Development

#### CSR and Behavioral Outcomes (Customer Citizenship Behavior)

Customer citizenship behavior (CCB) has been an area of augmented interest in service literature (Yi et al., 2013), which is discretionary and covers actions such as providing feedback, assisting other customers and making suggestions for improvement (Bettencourt, 1997; Groth, 2005; Lii and Lee, 2012). Past studies are scant in providing organization-specific factors in predicting CCB (e.g., Tung et al., 2017; Zoghbi-Manrique-de-Lara et al., 2017; Choi and Lotz, 2018), and in particular, the role of CSR has largely been ignored.

The said association can be explained with the Affect Infusion Model (AIM; Forgas, 1995), which proposes that one’s emotions influence cognitive judgments, here CSR is assumed to influence the emotions of customers positively, and in return influencing customers’ response and behavior. It is observed that CSR influences customers’ emotions positively (Lee and Yoon, 2018) and makes them respond with positive attitudinal and behavioral responses (Plewa et al., 2015). Here CCB being the discretionary behavior, is assumed to be influenced by high emotions toward a brand, which is determined by fir CSR activities (Xie et al., 2017; van Tonder et al., 2018). Similarly, AT also predicts the association of CSR and CCB by proposing that one may develop a bond with the product/service provider and reciprocate positively (e.g., CCB being the response). Past studies have witnessed that CSR investments influence customers as they show love and affection toward the brand/firm (Rodrigues and Costa, 2017). Attribution theory (Heider, 1958) undertakes that individuals attribute their actions to internal or external forces. As CSR is an external positive force (attribute) it is expected to influence customers’ behaviors positively (García-Jiménez et al., 2017). Based on the given theoretical premise following association is assumed:

H1: Firm CSR activities and customers’ CCB are positively related.

#### CSR Relation With Behavioral Outcomes Through Perceptual and Attitudinal Factors

This study also sought to test the mediation mechanism between CSR and CCB through perceptual (service quality) and attitudinal (affective commitment) factors. As discussed earlier, CSR being the external attribute may influence the emotions (Xie et al., 2017; van Tonder et al., 2018), attitude and behavior of customers (Rodrigues and Costa, 2017). So we assume that CSR will positively influence customers’ emotions and they will feel positive about firms and services (service quality) and depict a positive attitude (i.e., affective commitment). Literature focuses on CSR and service quality as independent constructs predicting the same outcomes (e.g., He and Li, 2011; Kim and Kim, 2016), but the link between them is rarely investigated. Out of the few studies, Poolthong and Mandhachitra’s (2009) study highlighted that customers’ have a positive view about bank’s CSR investment increases in their perceptions of service quality, while no such study has focused on hospitality and the food industry. We also assumed that CSR will positively influence customers’ perceptions of service quality. Similarly, past studies have noticed that CSR positively influences customers’ attitudinal responses (McDonald and Hung Lai, 2011; Jarvis et al., 2017), for instance, trust, brand identification (He and Li, 2011), brand image and loyalty (Kim and Kim, 2016; Servera-Francés and Piqueras-Tomás, 2019). Here affective commitment being an emotional attachment and attitudinal response (Izogo, 2017), may be influenced by the CSR activities.

We also entailed the investigation of the relationship between service quality perceptions, affective commitment and citizenship behavior of customers, which has largely been unattended in the past, but some other determinants have been reported in the past. For instance, commitment is predicted by brand experience (Johnson et al., 2008; Iglesias et al., 2019). Similarly, Fernandes and Pinto (2019) highlighted that customers’ quality of interaction with service employees increases their commitment level. They also found that such customers praise service providers to others, which is an important dimension of CCB. It is also observed that provision of better services make customers feel a sense of ownership toward the firm (Béal and Sabadie, 2018), influence them at an emotional level (Aurier and Séré de Lanauze, 2012; Izogo, 2017; Islam et al., 2019), and this ultimately influences their commitment and behavior toward the firm (Ranganathan et al., 2013; Rai and Nayak, 2019). Affect infusion model (Forgas, 1995) and AT (Bowlby, 1969) can also predict this association. These theories assume emotions influence judgments of customers (Forgas, 1995) and create a bond with sources of emotions (Bowlby, 1969). Service quality is the source of emotions that may create a psychological and emotional bond (i.e., commitment) which allows customers’ to make judgments and decide on behavior (i.e., CCB).

While linking customer commitment and citizenship behavior, it is observed that their attitude influences behavior, for instance, customer satisfaction (Anaza, 2014) and, commitment (Choi and Lotz, 2018; van Tonder et al., 2018). Attribution theory (Heider, 1958) can explain this relation, as CCB could be attributed to the internal or external factors, here customer commitment is a potent internal force. Based on this premise we also assume that both CSR and the service quality of customers’ belief about an organization which (if positive) may influence customers’ affective commitment that can lead to high CCB.

The aforementioned discussion helped us assume the link between CSR, service quality, affective commitment, and CCB which is assumed through serial mediation mechanism. The mentioned associations could be based on the AT (Bowlby, 1969) and attribution theory (Heider, 1958). Attachment theory proposes that customers may have a bond with the firm which is based on cognitive, emotional and social developments. Attribution theory provides sources of emotional relations by proposing internal and external forces being the attributions. CSR, as an external force, is found to influence emotions positively which in turn influences perceptions about service quality and affective commitment.

The value of such mechanisms is valued by past studies, for instance, Xie et al. (2017) commented that CCB association with predictors may better be explained by some explanatory mechanism. Zoghbi-Manrique-de-Lara et al. (2017) study reported that management treatment of customers influences services quality perceptions which in turn influences CCB. Thus helping us assume that firm CSR investment influences customer CCB positively. CCB has also been predicted by contextual factors (Cheng et al., 2016), while CSR could also be deemed as such a factor. On the other hand, Anaza (2014) highlighted the role of both personal and organizational factors in predicting CCB, thus both CSR and service quality can predict CCB. Tung et al. (2017) highlighted the need for future studies focusing on the effects of repeated interactions on CCB, as such interactions have lasting effects on customers. Based on this and previous sections mentioning link among variables of interest it is assumed that the relationship between CSR and CCB is explained by both service quality perceptions and affective commitment of customers, which is hypothesized as follows:

H2: CSR and CCB positive relations are mediated by both service quality perceptions and affective commitment such that the relation is in serial mediation.

## Research Methodology

The hypothesized model data was collected from customers of restaurants between April – September 2019 from two major cities of Pakistan covering Islamabad (the federal capital) and Lahore (the provincial capital of the largest province of the country). The data was collected through personally administrated questionnaires. 1,500 questionnaires were floated to the customers of 259 restaurants, while only 994 complete responses were used for analysis. The respondents included 59 male, 83% university students, 88% unmarried, and 89% regular visitors. The average age of respondents was 20.94 years. The sample could be considered useful as it has been witnessed that the hoteling trend is increasing in youth i.e., Millenials (Islam et al., 2019; Ahmed et al., 2020). The sampling technique was convenienient and was self-sampling (volunteer responses). Both the techniques have been widely used for unknown population and in marketing literature. To get better and fair results, data collection was done when the customers were dining at the selected restaurants, for which permission was taken from the management of the restaurants.

The measures used in this study are well-established and have been widely used in the past. For instance, CSR was measured using Brown and Dacin’s (1997) four items scale. The exemplary items are “I believe that this restaurant acts responsibly against obesity issues.” The affective commitment was measured using Mende and Bolton’s (2011) three items scale which covered items such as “I enjoy being a customer of this restaurant.” Service quality was measured through two dimensional (physical quality & staff behavior) using the scale of Ekinci (2001) and Ekinci et al. (2008) covering three and four items, respectively. These dimensions covered statements such as “The restaurant is tidy” and “the staff of the restaurant is helpful and friendly.” Customer citizenship behavior was measured using Yi and Gong’s (2008) six items scale. The results of data analysis are shown hereafter. It contained items such as “I would say positive things about this restaurant to others.”

The data collected through the questionnaire was analyzed using Statistical Package for Social Sciences (SPSS) and Analysis of Moment Structure (AMOS), this is an exstensivelly used statistical software. The analysis was carried out using descriptive statistics, Structural Equation Modelling (SEM) and Hayes process macros (Hayes et al., 2017). The use of SEM has been widely accepted in social and management sciences because it uses both structural and measurement simultaneously (Hair et al., 2010).

## Findings

### Measurement Assessment

Table 1 contains descriptive statistics and reliability results. The measures of the study were assessed on five points Likert scale. The mean scores reported in the table highlight that all the means are close to the score of four, which denoted the response of “Agree.” Moreover, the reliability values, assessed through Cronbach Alpha, are (0.89–0.93) and were also well above the threshold value. It is found in the literature that reliability value of 0.65–0.80 is considered adequate for scale measuring variables of human aspects (Green et al., 1977; Vaske, 2008). Thus the scales were considered to be reliable.

**TABLE 1:** Descriptive statistics.

**Study variables**	**Cronbach alpha**	**Descriptives**	
		**Mean**	**SD**
Perceived CSR	0.91	3.958	0.905
Service quality perceptions	0.89	4.320	0.692
Affective commitment	0.93	3.890	0.852
Customer citizenship behavior	0.90	3.840	0.849

Tables 2 and 3 cover the results of the adequacy of measures, which was assessed through confirmatory factor analysis (CFA) (Anderson and Gerbing, 1982; Hair et al., 2010). The table covers the values of the factor loading, average variance extracted and composite reliability. It is evident that all the items loaded well on their respective constructs as all the values are well above the threshold value of 0.60, thus the measure was assumed to be adequate. The reported values were also used to assess convergent validity which was assumed to be present, as the values of AVE were well above the threshold values of 0.5 (Fornell and Larcker, 1981; Iglesias et al., 2019). It is thus to assume that the convergent validity was present and scales met the validity assumptions. Discriminant validity was further assessed by comparing the bivariate correlation among constructs and each construct AVE square root (Hair et al., 2010). As all values exceeded the minimum limit, it was assumed that the discriminant validity was held (Table 3).

**TABLE 2:** Confirmatory factor analysis.

**Construct**	**Item**	**Loading**	**CR**	**AVE**
Perceived CSR	This restaurant is considering customers’ health	0.74	0.91	0.90
	This restaurant acts responsibly against obesity issues	0.88		
	This restaurant has a sense of responsibility to customer’s health	0.94		
	This restaurant is socially responsible	0.79		
Service quality	The décor of the restaurant is beautifully coordinated with great attention to details	0.80	0.90	0.82
perceptions	The restaurant is tidy	0.79		
	The restaurant provides a comfortable room	0.83		
	The staff of the restaurant is helpful and friendly	0.89	0.89	0.79
	The staff of the restaurant seems to anticipate what I want	0.88		
	The staff of the restaurant listens to me	0.76		
	The staff of the restaurant is talented	0.89		
Affective	I enjoy being a customer of this restaurant	0.79	0.90	0.77
commitment	I have positive feelings about this restaurant	0.84		
	I feel attached to this restaurant	0.90		
Customer	I would say positive things about this restaurant	0.89	0.89	0.80
citizenship	I would give constructive suggestions to this restaurant on how to improve its services	0.80		
Behavior	When I have a useful idea on how to improve service, I would communicate it to someone in this restaurant	0.91		
	When I experience a problem in this restaurant, I would let someone know so that they can improve the service	0.87		
	I would do things that make the employees’ job easier	0.79		
	I would carefully observe the rules and policies of this restaurant	0.88		

**TABLE 3:** Discriminant validity.

	**PCSR**	**SQ**	**AC**	**CCB**
PCSR	**0.81^a^**	–	–	–
SQ	0.63^b^	**0.79**	–	–
AC	0.60	0.68	**0.94**	–
CCB	0.59	0.58	0.69	**0.89**

*^*a*^AVE square root in diagonal. ^*b*^Bivariate correlation among constructs.*

### Common Method Biasness

As the study was one-spot in nature and data was self-reported, we sought to verify the data for the possible presence of common method variance (CMV) by using Harman’s single-factor test (Podsakoff et al., 2003). We observed that a single factor only accounted for 32.87% variance (<50%) thus helping us assume that the CMV was not severe (Podsakoff et al., 2003). Moreover, CMV was also assumed not to be severe as the correlation values were below threshold (i.e., <0.9; Pavlou et al., 2007).

### Results of Hypotheses Testing

Hypotheses testing results are shown in Table 4. The table contains the results of both the hypotheses. At first instance, the result of the direct relationship between service quality and customer citizenship is reported. It is evident from the table that CSR is significantly and positively related with CCB (0.3985; *p* < 0.001), which helps us infer that when restaurants invest in the CSR activities, customers respond with positive behavior beyond their traditional roles (extra-role behavior – CCB), thus H1 is supported.

**TABLE 4:** Path analysis results.

**Hypotheses**	**Path**	**Estimate**	**SE**	***p***	**CI**	**Result**
H1	CSR–CCB	0.3985	0.014	0.0000	[0.339; 0.440]	Supported

**Hypotheses**	**Path**	**Estimate**	**Boot SE**	**Boot LLCI**	**Boot ULCI**	

H2	PCSR-SQ-AC-CCB	0.1613	0.0063	0.0021	0.0263	

*CSR, corporate social responsibility; CCB, customer citizenship behavior; SQ, service quality; AC, affective commitment.*

As this study also entailed that the investigation of an indirect relationship between CSR and CCB through serial mediation of service quality and affective commitment, the remaining part of the table covered the results of serial mediation mechanism. The findings highlight that the relationship between CSR and CCB through the serial mediators (service quality and affective commitment) is significant (0.1613, Boots SE 0.0063). The results proved to be significant as there was no presence of non-zero and the signs of both ULCI and LLCI were positive (LLCI 0.0021 and ULCI 0.0263). These results helped us conclude that H2 was also supported. The results thus explain that the CSR efforts of a restaurant will cause improved service quality perceptions and affective commitment of customers and in turn, their citizenship behavior will upsurge.

## Discussion and Conclusion

This study has focused on investigating the perceptual (service quality), attitudinal (affective commitment) and behavioral (customer citizenship behavior) outcomes of CSR. The need for this study had been highlighted and called up by past studies (e.g., Xie et al., 2017; Zoghbi-Manrique-de-Lara et al., 2017; Choi and Lotz, 2018; Islam et al., 2019). For instance, Engizek and Yasin (2017) invited researchers to investigate the attitudinal and behavioral outcomes of CSR in tandem. While investigating the predictors of CCB, Choi and Lotz (2018) invited future researchers to focus on contextual factors. CSR being the contextual factor, is found to influence CCB significantly.

The study also entailed the investigation of mediation mechanism between CSR and CCB through service quality and affective commitment, which has also been directed by past studies. For example, Tung et al. (2017) commented that CCB studies should focus on repeated interactions and psychological mechanisms predicting it. Islam et al. (2019) also valued such mechanism, as they commented that service quality outcomes should cover both attitudinal and behavioral variables in a mechanism. Our findings proved that CCB is influenced by a mechanism (through a serial mediation), which covers perceptual, attitudinal and behavioral variables. These findings were in-line with past studies; for instance, Jarvis et al. (2017) also reported that CSR influences customers’ emotional, attitudinal and behavioral responses. The emotional and attitudinal outcomes have also been investigated in other studies as well (e.g., Poolthong and Mandhachitara, 2009; He and Li, 2011; McDonald and Hung Lai, 2011; Kim and Kim, 2016; Fernandes and Pinto, 2019; Iglesias et al., 2019; Servera-Francés and Piqueras-Tomás, 2019). Béal and Sabadie (2018) and Izogo (2017) also found that taking care of society and customers, influences customers and they generate positive perceptions that ultimately affects their response.

These findings support the hypotheses, past studies and theoretical premise of the study. The findings prove that customers attribute their behavior to external factors (here CSR; attribution theory, Heider, 1958). While CSR could also be presumed to be a force that may cause a bond between customers and a firm, and can make them reinforce relationships (CCB; AT, Heider, 1958). The said association also supports presumptions of the affect infusion model (Forgas, 1995) which proposes the value of emotions in making judgments and responses. Here it is observed that CSR works at an emotional level and influences judgments (perceptions and attitudes – service quality and affective commitment) and responses (behavior – CCB).

The findings discussed in the highlight of the aforementioned section show that this study entails some novel explanations linking CSR with CCB through perceptual and attitudinal factors as a serial mediation mechanism. Past studies have not linked CSR and service quality, rather investigated them as independent variables in the models (e.g., He and Li, 2011; Kim and Kim, 2016). The serial mediation model was supposed using theoretical triangulation, i.e., affect infusion model (Forgas, 1995), attachment (Bowlby, 1969) and attribution theories (Heider, 1958), and the findings of the study stand tall with the theoretical assumptions made in these studies.

Additionally, this study adds value by generating a useful message for the managers of restaurants, due to increased competition in the food industry it has become imperative for management to generate loyal customers having a high level of citizenship behavior (Islam et al., 2019; Ahmed et al., 2020). While looking at the ways of increasing CCB it has been suggested that customers should be given employee like treatment. The study offers a novel explanation to the managers of the restaurants, as they can improve the CCB by increasing the CSR investments. Moreover, this study provides a complete mechanism that covers perceptual, attitudinal and behavioral outcomes offered by CSR investments. Although CSR has been widely recognized as a tool to boost profits and employees’ responses, the outcomes focusing on the customers’ responses have not been managerially evaluated (Huang et al., 2019).

Though this study assumes and tests a novel mechanism and offers wider implications, it is still prone to some limitations. The foremost is the use of cross-sectional design, as it was not possible to approach customers with intervals (for lag or longitudinal study). Though the CMV was not a severe yet longitudinal design, it may offer better results. The sample of the study consisted of millennials only while the other age cohorts may offer different results. This study also entails the investigation of service quality through only two dimensions (i.e., physical quality and staff behavior), while it has been investigated through tangible and intangible service factors which could be an important consideration for future studies. As this study covers attitudinal and behavioral outcomes, future studies may focus on other variables (e.g., engagement, patronage intentions, WOM). Future studies could also investigate CCB as a multidimensional construct and evaluate them independently.

## Data Availability Statement

The raw data supporting the conclusions of this article will be made available by the authors, without undue reservation, to any qualified researcher.

## Author Contributions

IAh, MN, IAl, and FA contributed in: definition of research objectives, developing models, hypotheses, data analysis plan, article writing, revision/proofreading, and final approval. AK and MS contributed in data collection, analysis, drawing limitations, future directions and conclusion of the study.

## Conflict of Interest

The authors declare that the research was conducted in the absence of any commercial or financial relationships that could be construed as a potential conflict of interest.
